# Challenges and opportunities of complexity theory in health care systems

**DOI:** 10.1016/j.jtumed.2025.06.006

**Published:** 2025-07-03

**Authors:** Ashraf A'aqoulah, Ashraf A. El-Metwally, Bader Al Khateeb, Duaa Alammari, Awad Alshahrani, Aljohara Aldubikhi, Abdulrahman Alsulami, Faris Fatani, Nisreen Innab

**Affiliations:** a Health Systems Management Department, College of Public Health and Health Informatics, King Saud Bin Abdulaziz University for Health Sciences, Riyadh, KSA; b Office of Research, King Abdullah International Medical Research Center, Ministry of National Guard Health Affairs, Riyadh, KSA; c Epidemilogy and Biostatistics Department, College of Public Health and Health Informatics, King Saud Bin Abdulaziz University for Health Sciences, Riyadh, KSA; d Family Medicine Department, College of Medicine, King Saud Bin Abdulaziz University for Health Sciences, Riyadh, KSA; e Internal Medicine Department, King Saud Bin Abdulaziz University for Health Sciences, Riyadh, Saudi Arabia; f Department of Medicine, Ministry of National Guard-Health Affairs, Riyadh, KSA; g Public Health Department, Saudi Electronic University, Riyadh, KSA; h Population Health Management Department, Riyadh Second Health Cluster, Riyadh, KSA; i Department of Computer Science and Information Systems, College of Applied Sciences, AlMaarefa University, Riyadh, KSA

**Keywords:** Challenges, Complexity theory, Health care system, Opportunities, نظرية التعقيد, نظام الرعاية الصحية, التحديات, الفرص

## Abstract

Recent studies of health and complexity have proposed a new definition of health that encompasses the systematic nature of health in order to depict its inherent complexity. The interaction between a clinician and patient has many of the characteristics of a complex adaptive system. The complex nature of primary health care systems, the complexity of real-time data analytics in health care using information systems, and complexity related to the prescription of drugs further reflect the complexity of health care systems. These characteristics indicate that it is necessary to transform health care from an egocentric system to an ecocentric system by using a complex network electronic knowledge research model that provides community engagement in decision making for health improvement and involves adaptive leadership implementing different strategies for solving complex problems. Indeed, it is essential to use a transdisciplinary approach to redesign the health system by developing effective monitoring and evaluation systems inside health systems to ensure patient safety and information transparency, and the effective use of information technology to improve the quality of the services delivered.

## الملخص

اقترحت الدراسات المنشورة مؤخرًا حول الصحة والتعقيد تعريفًا جديدًا للصحة يشمل الطبيعة المنهجية للصحة لتصوير التعقيد الكامن في الصحة. يوضح التفاعل بين الطبيب والمريض العديد من خصائص النظام التكيفي المعقد. تعكس الطبيعة المعقدة لنظام الرعاية الصحية الأولية، وتعقيد تحليلات البيانات في الوقت الفعلي في الرعاية الصحية باستخدام أنظمة المعلومات، والتعقيد المحيط بوصف الأدوية المزيد من التعقيد في نظام الرعاية الصحية. تتطلب هذه الجوانب تحويل الرعاية الصحية من نظام يركز على الخدمة إلى نظام يركز على البيئة، باستخدام نموذج بحثي معقد للمعرفة الإلكترونية للشبكة يوفر مشاركة المجتمع في صنع القرار لتحسين الصحة وينطوي على قيادة تكيفية تنفذ استراتيجيات مختلفة لحل المشاكل المعقدة. في الواقع، من الضروري استخدام نهج متعدد التخصصات لإعادة تصميم النظام الصحي من خلال تطوير أنظمة مراقبة وتقييم فعالة داخل الأنظمة الصحية، وسلامة المرضى، وشفافية المعلومات، والاستخدام الفعال لتكنولوجيا المعلومات لتحسين جودة الخدمات المقدمة.

## Introduction

Providing health care services is a complex task because different systems interact with each other. For example, many studies have described the relationship between physical health and mind.^1^^,^^2^ Other studies have also demonstrated the influence of expectations on treatment outcomes,^3^^,^^4^ and show how lifestyle and diet affect well-being.^5^^,^^6^

In this study, we identify the challenges that affect health systems in the context of using complexity theory to represent the complex nature of health care systems. We also highlight opportunities for health systems in the context of complexity theory by aiming to improve the quality of health care services.

## Challenges for health systems in the context of complexity theory

### Health is a complex phenomenon

It has been argued that the concept of health is not as simple as the absence of disease, but instead it can be defined by understanding that health has different dimensions and each dimension is potentially a separate system.^7^ Therefore, the notion of maintaining good health at the level of an individual can be understood as a complex and adaptive system.^8^ Over time, the definition of health has become quite complicated. In particular, when defining health, it is important to consider its experiential, physical, and behavioral dimensions. In the last 100 years, around 14 different models have been developed to try to define health. At present, research is focused on describing health as the product of a larger system composed of societal and individual levels of organization, and its relationship to the external environment.^9^ This and many other explanations of health have implications for health, health care, and the provision of health care services. In this context, recent studies of health and complexity have proposed a new definition of health that encompasses the systematic nature of health and tries to depict the inherent complexity of health.^10^ Therefore, health can be defined as a balanced state between physical, emotional, social, and cognitive sense-making domains. In any local environmental context, a health state exists within a multidimensional phase space of physical integrity, functional performance, and subjective experience to produce an entropic state that is most consistent with viability.^11^ Health is influenced by a wide array of social, economic, and environmental determinants, making it difficult to design one-size-fits-all policies. This complexity creates space for tailored, community-specific interventions that leverage local data and stakeholder engagement.

### Complexity during consultation with a clinician

The interaction between a clinician and patient has many of the characteristics of a complex adaptive system. The interactions between different agents during consultations are quite diverse, thereby resulting in the phenomenon of self-organization and emergence in the form of healing of the patient. The phenomenon of healing is quite unpredictable and related to changes based on many important factors, including clinical wisdom, emotional self-management, projection of confidence in use of medical technology, and mindfulness. Clinicians need to decide when to introduce novelty into the care process to convert unproductive interactions into interactions that help the patient to heal.^12^ Studies have investigated specific conditions such as diabetes to help us understand the complexity of managing these condition so we can effectively plan to provide the requisite services to patients.^13^ In particular, behavioral problems such as intimate partner violence need to be viewed through the lens of complexity science because of their heterogeneous, multicausal, and dynamic nature.^14^ Clinical consultations often involve navigating multifaceted patient concerns, which can overwhelm both the clinician and the patient, potentially leading to incomplete assessments. This complexity presents an opportunity for adopting holistic, patient-centered care models and fostering interdisciplinary collaboration.

### Complexity in the primary health care system

The traditional reductionist approach to primary health care focuses on attaining some vaguely defined goals by emphasizing performance indicators and capturing process indicators, thereby giving the impression that these are some of the most important characteristics of primary health care. This approach may reduce primary health care to less than sum of its parts.^15^ Previous studies have demonstrated that substantial variation occurs in the provision of care during primary health care but this variation might provide an opportunity for adapting to the diverse needs of the community. When designing interventions to improve primary health care, it is important to identify different factors and use tools specified in complexity theory. At a minimum, it is important to understand the key factors at the level of interaction between the care giver and community members, and at the level of the organization.^16^ EQUIP was designed as an intervention to provide equity-oriented care in primary health care settings after comprehensively studying various factors, such as the client population, staff characteristics, organizational milieu, policy and funding environment, and geographic milieu.^17^ This is a good example of how various contextual factors can be studied in the primary health care setting and then used to design interventions to obtain the intended outcomes. Another example is using the Meikirch model as a new definition of health that encompasses complex adaptive systems to provide a better understanding of patients' diseases and their treatment in primary care.^18^ Primary health care systems often face layered challenges, such as high patient volumes, limited resources, and workforce shortages, and these complexities can lead to inefficiencies. This complexity creates an opportunity to invest in system integration and digital health infrastructure.

### Complexity and real time data analytics in health care

Many monitoring devices are used for managing patients, and they continuously generate a high volume of variable data.^19^ The data generated by these devices need to be managed and effectively shared to help formulate actionable insights in many health systems, but this involves many factors, thereby further illustrating the inherent complexity of health system.^20^ These sources of data can be converted into complex but useful decision support systems to help clinicians make personalized clinical decisions and reduce errors when providing clinical care to patients. The infrastructure of these information systems is complicated but understood by relevant experts, although the practical implementation of these systems is complex and can become chaotic at times due to problems such as security breaches. Therefore, it is important that the relevant professionals who manage these systems have a comprehensive understanding of complexity theory and systems thinking. The health care environment generates vast amounts of data from diverse sources. It is challenging to manage, integrate, and analyze these data in real time to support clinical decision making. This complexity provides a critical opportunity for investing in advanced analytics platforms and artificial intelligence to process real-time data streams in an efficient manner.

### Complexity of drug prescription

Decisions related to the prescription of medicines can be quite complex and contingent upon several factors.^21^ An important factor that influences the prescription of medicines by physicians is the fact that decisions regarding making prescriptions are influenced by patients and their perceptions. In addition, the prescription pattern during an informal interaction may be affected by health staff and relatives of the patient. The main aim of the physician is to treat the patient and prescribe them medicine during the consultation process, but many other factors might be important, including the physician's financial goals. Physicians are overloaded with information from various scientific and non-scientific sources,^22^ but most physicians lack a set system or protocols to filter relevant and scientifically robust sources of information, resulting in confusion. This information overload is complicated by uncertainties due to several issues, including imperfect reporting by the patient, uncertainty about the correct diagnosis, the effect of drug on a specific patient, carry over effects of drugs, and less than perfect information regarding other drugs that the person might be taking. The price of the drug remains one of the key factors when deciding whether a specific drug can be prescribed to a patient. In many cases, decisions are made based on affordability for a patient rather than following the clinical guidelines. Advertisement efforts play important roles in the physician's decision-making process. The prescription pattern is complicated even further when we consider the gifts given directly to physicians or to help physicians in different ways, such as sponsorship for conferences and other personal travel.^23^ Given this context, it is clear that drug prescription is quite a complex phenomenon. Any decision related to managing the prescription patterns of physicians will require an understanding of the complexity of the matter and using suitable tools to improve the situation. Prescribing medications involves navigating a complex array of factors, including polypharmacy, patient-specific conditions, and patient adherence. This complexity increases the risk of errors and adverse drug events. This complexity presents an opportunity to integrate clinical decision support systems into prescribing platforms, which can provide real-time alerts about interactions and dosage adjustments.

## Opportunities for health systems in the context of complexity theory

### Complex network electronic knowledge research (CoNEKTR) model

This opportunity is aligned with complexity and real-time data analytics in health care. The CoNEKTR model facilitates collaborative, real-time data use and knowledge translation across care environments by effectively integrating concepts related to systems thinking and complexity theory to solve problems associated with health promotion. This model helps to effectively engage various relevant groups to ensure effective and sustained change efforts. The CoNEKTR model is particularly effective for engaging communities to ensure participatory decision making and improve the health of the community. The CoNEKTR process has several steps, including gathering the group, giving an introduction to the topic under discussion, brainstorming to collectively obtain inputs and formulate ideas to solve relevant health problems, analyzing the collected qualitative data, further discussions within groups around different themes that emerge from the generated data, planning for the future based on these discussions, posting ideas online to obtain feedback from the wider community, generating online discussions on topics, and finally identifying action items and activities to work toward solutions for different health problems. This outline appears quite simple but it uses several concepts from systems thinking and complexity theory,^24^ including the following.

a. When convening the group, diversity should be retained to ensure maximum variation in the collected qualitative data.
b. Discussion in groups provides an opportunity to understand the existing social networks relevant to the problem under consideration.
c. Introducing the existing knowledge during discussions and considering path dependencies.
d. Specifying a problem and determining boundaries for the specific problem.
e. Encouraging the generation of novel solutions to the problem at hand by using catalytic questions to drive generative thinking.
f. Emergent patterns are identified in the system during data analysis.
g. Forming the research group around thematic areas identified in the process encourages self-organization.
h. Presenting the ideas generated by each group to other groups in order to provide feedback about ideas. Similarly, feedback is also obtained after sharing information online with other people.

### Adaptive health leadership

This opportunity is aligned with complexity during consultation with a clinician by supporting clinicians when navigating uncertainty, emotional labor, and multi-dimensional decision-making in patient encounters. Adaptive leadership focuses on the part of the operation that is important to the outcome of interest and omitting other aspects without which the organization can function well and effectively.^25^ In addition, this leadership approach focuses on the root causes of different problems to ensure that scalable and sustainable solutions are identified. An important characteristic of an adaptive leader is adaptation based on formal and informal feedback received.^26^ An adaptive leader is prepared to take calculated risks and experiment with different strategies to ensure adoption of new novel approaches for solving complex problems.^25^ This concept argues that leadership is a personal capacity or capability and not contingent upon the formal role of an individual in a health care organization or system. According to the adaptive leadership concept, a health care professional can adopt the role of a leader when providing health care services to a patient. The adaptive leadership framework was formulated based on complexity science concepts. In this framework, the patient at the point of care is affected by two major types of challenges: technical challenges and adaptive challenges. Technical challenges are not too complicated to address and the subjective expertise of clinicians is required to make decisions on behalf of the patient.^27^ Adaptive challenges require the patient to modify their behavior in response to a chronic condition, including greater self-discipline by patients, such as by modifying their lifestyles. The role of adaptive leadership by clinicians involves providing technical support and helping with decision making, as well as working as a leader by mobilizing the patient and their care givers to adapt to the new lifestyle that is needed, thereby increasing the patient's ability to handle their challenges.^25^ This role requires that clinicians are empathetic to the patient and they must try to understand the situation exactly as the patient or their caregiver sees it. This framework appears simple in theory but its implementation is quite complex. Therefore, optimizing the health provider's leadership role so it is suitable for different situations is an active area of research.

### From egocentric health system to ecocentric health systems using theory U

This opportunity is aligned with health as a complex phenomenon, where the focus is on shifting mindsets and structures toward interconnected, adaptive systems that embrace the complexity of health. This theory helps planners and decision makers to let go of different decisions made in the past and focus on the future, allowing transformation from an organization that is egocentric to an organization that is focused on an ecosystem.^28^ The process of letting go of the past and planning for the future involves a number of transformative stages, include suspending, observing, sensing, crystallizing, prototyping, and realizing. This process also focuses on planning for the future and letting new opportunities emerge by having an open mind to new opportunities, as well as trying to understand the stakeholder's perspective with an open heart and having a clear will to commit to actualizing the change process.^29^ This concept focuses on an approach where decision makers let go of things that are not essential for the vision of the organization. In many cases, these issues are related to the ego of the decision maker, and thus it is important to let go of the ego in order to formulate and adopt solutions that are needed to produce a better ecosystem. After letting go of the old processes and functions, it is time to welcome new ideas, and enact and embody them.^28^ This process allows decision makers to get in touch with their own self and work more effectively toward the vision of the organization. It also helps decision makers to improve their leadership capabilities.

### Using a transdisciplinary approach to redesign the health care system

This opportunity is aligned with considering complexity in the primary health care system by promoting integration across sectors and disciplines to rebuild a primary care system with responsiveness and resilience. According to the American Institute of Medicine, around 98,000 individuals die each year due to errors that occur in hospitals.^30^ Importantly, it was shown that these errors could not be attributed to any specific team member, but instead they were systematic errors, and thus the whole system needs improving. Given this issue, a transdisciplinary approach is required to rethink and redesign the way we approach health and health care.^31^ The purpose of all health organizations should be to reduce the burden of illness, disability, and injury by making health care services safer, more effective, patient centered, timely, efficient, and equitable. The development of effective monitoring and evaluation systems inside health systems is recommended.^32^ Health services should be customized according to the needs of the patients. Furthermore, an important recommendation is that the safety of patients should be a key defining property of the health system rather than assuming it is optional. Transparency of information to patients is also recommended to help them make informed decisions in the context of their conditions.^33^ The effective use of information technology is essential to improve the quality of services delivered.^34^ Hand written data should be eliminated in order to reduce errors.

### Direction for health care systems

This opportunity is aligned with considering the complexity associated with drug prescription by encouraging system-level guidance and policy frameworks to support safer, evidence-based, and coordinated prescribing practices. It is important for health systems to reduce the lag in innovation to improve the quality of service provided in the context of complex health care systems.^35^ It is also necessary to build the capacity of relevant health professionals to ensure the effective adaptation of innovative technologies. To manage health systems effectively, it is important to have end-to-end interoperability among different electronic health records, thereby ensuring that informed decisions are made in a timely manner. A culture of sharing data will ensure the generation of contextual evidence and proactive steps are required to accelerate the process that generates evidence.^36^ Regulatory frameworks must be patient oriented. Strategies should be optimized to design and then integrate decision support systems into routine health delivery systems.^37^

In the era of data science and big data, it is possible to provide personalized care to each patient in hospitals as well as the general population, which necessitates proactively working toward applying and adapting delivery systems that are focused on personalized medicine. Research is needed into building the capacity of health professionals to ensure the formulation of a continuous learning health system. Capturing and using data with different machine learning models can assist with decision making.^35^ Machine learning and artificial intelligence have the potential to improve the service delivery infrastructure. Research insights should be scaled up to ensure timely action.

## Conclusion

Challenges ranging from the complexity of clinician consultations and primary health care systems to the intricacies of real-time data analytics and drug prescription are not isolated, but instead they are deeply interdependent. These issues are mutually reinforcing, creating a cycle of inefficiency and risk that undermines patient outcomes. However, the proposed opportunities such as adopting holistic and interdisciplinary care models, investing in digital health infrastructure, integrating advanced data analytics, and implementing decision support tools are not merely targeted fixes, but instead they offer a complementary framework that enhances system-wide coherence.

In the current context, understanding based on systems thinking and complexity theory is important for all health professionals, but especially health leaders. It is important to gain the requisite conceptual understanding of relevant theories, methods, and tools in order to effectively make decisions in health care at all levels. Finally, the current era of health data science and availability of various machine learning algorithms presents new opportunities and challenges that are relevant to solving complex systematic problems in health care.

## Patient and public involvement

Patients and/or the public were not involved in the design, conduct, reporting, or dissemination plans for this research.

## Patient consent for publication

Not applicable.

## Authors contributions

All authors made the same contribution. All authors have critically reviewed and approved the final draft and are responsible for the content and similarity index of the manuscript.

## Ethical approval

All relevant data are within the manuscript and Supporting Information files.

## Data availability statement

Not applicable.

## Source of funding

No funding was obtained to conduct this research.

## Conflict of interest

The authors have conflicts of interest to declare.
